# Electroencephalography-based monitoring of delirium in the ICU: what are the opportunities?

**DOI:** 10.1186/cc10945

**Published:** 2012-03-20

**Authors:** AW Van der Kooi, FS Leijten, RJ Van der Wekken, AJ Slooter

**Affiliations:** 1 University Medical Center Utrecht, the Netherlands

## Introduction

Up to 80% of patients experience delirium during their ICU stay [1,2]. The most sensitive screenings tool for delirium in a research setting is the Confusion Assessment Method for the ICU (CAM-ICU), but the low sensitivity of the CAM-ICU in daily practice (47%) hampers early detection of delirium and thereby delays treatment [3,4]. Therefore, there is a need for an objective tool for continuous delirium monitoring. Diagnosis of delirium can also be conducted using electroencephalography (EEG) [5]. EEG with a limited number of electrodes and automatic processing may be a more sensitive approach for delirium monitoring. The aim of this systematic review is to explore opportunities for automatic detection of delirium by summarizing EEG characteristics of delirium.

## Methods

A systematic literature search was conducted in Embase and Medline. Articles concerning quantitative EEG and delirium were included. Per article, the differences between delirious and nondelirious subjects in EEG characteristics were noted.

## Results

Fourteen studies were included, which were predominantly conducted in older patients. The relative power of the theta frequency band was most often and without exception significantly different (7/14 studies) in delirious subjects. Other frequently measured parameters were the relative power of the delta and alpha frequency band and the peak frequency. None of these studies addressed the optimal electrode deviation or the question of how to distinguish sleep from delirium.

## Conclusion

Given the feasibility for continuous EEG monitoring in ICU, EEG delirium monitoring in ICU patients seems to be promising.
